# Interplay of human gastrointestinal microbiota metabolites: Short-chain fatty acids and their correlation with Parkinson’s disease

**DOI:** 10.1097/MD.0000000000037960

**Published:** 2024-04-26

**Authors:** Jiaji Liu, Qi Chen, Ruijun Su

**Affiliations:** a Inner Mongolia Medical University, Department of Laboratory Medicine, Affiliated Hospital of Inner Mongolia Medical University, Hohhot, China; b The Third Clinical Medical College of Ningxia Medical University, Ningxia, China.

**Keywords:** gut microorganisms, histone deacetylation, oxidative stress, Parkinson disease, short-chain fatty acids

## Abstract

Short-chain fatty acids (SCFAs) are, the metabolic byproducts of intestinal microbiota that, are generated through anaerobic fermentation of undigested dietary fibers. SCFAs play a pivotal role in numerous physiological functions within the human body, including maintaining intestinal mucosal health, modulating immune functions, and regulating energy metabolism. In recent years, extensive research evidence has indicated that SCFAs are significantly involved in the onset and progression of Parkinson disease (PD). However, the precise mechanisms remain elusive. This review comprehensively summarizes the progress in understanding how SCFAs impact PD pathogenesis and the underlying mechanisms. Primarily, we delve into the synthesis, metabolism, and signal transduction of SCFAs within the human body. Subsequently, an analysis of SCFA levels in patients with PD is presented. Furthermore, we expound upon the mechanisms through which SCFAs induce inflammatory responses, oxidative stress, abnormal aggregation of alpha-synuclein, and the intricacies of the gut-brain axis. Finally, we provide a critical analysis and explore the potential therapeutic role of SCFAs as promising targets for treating PD.

## 1. Introduction

Owing to risk factors including age, environment, and genetics, Parkinson disease (PD) has emerged as rapidly growing neurodegenerative disorder that, significantly impacts patients’ quality of life.^[1,2]^ Parkinson disease is characterized predominantly by the loss of dopaminergic neurons in the substantia nigra pars compacta and manifests as a neurodegenerative ailment. The key pathological feature of PD is the aggregation of alpha-synuclein (α-Syn) within neurons, leading to the formation of Lewy bodies.^[3]^ Simultaneously, from a pathological standpoint, neuroinflammation stands out as a primary contributing factor.^[4]^ The clinical manifestations of PD primarily include motor impairments characterized by stiffness, resting tremors, and bradykinesia, as well as non-motor disturbances dominated by hallucinations, depression, and dementia.^[5,6]^ Additionally, a myriad of prodromal symptoms, including constipation, nausea, and vomiting, are commonly observed in patients with PD, highlighting the prevalence of gastrointestinal reactions.^[7]^ These gastrointestinal functional disruptions have been acknowledged as crucial clinical symptoms since the early stages of PD research. Consequently, the gut microbiota PD association has attracted increasing research attention. Although the etiology of PD is unclear to date, there is widespread speculation that the microbial composition in the gastrointestinal tract and its metabolic byproducts may constitute pivotal factors influencing PD progression.^[8]^ This conjecture underscores the intricate interplay between neurodegenerative processes and the gut microbiome, opening avenues for further exploration into the mechanisms underlying the pathogenesis of PD.

The intestinal microbiota predominantly comprises bacteria, archaea, fungi, viruses, and other microorganisms parasitizing the gastrointestinal tract. Surpassing the number of human cells, these microorganisms carry a vast amount of genetic information, rendering them akin to an “organ” within the human body that engages with the host, in a symbiotic relationship. Bacteria, particularly genera such as Bacteroides and Firmicutes, constitute over 90% of the entire intestinal microbiota.^[9,10]^ This complex microbial community generates various metabolites that impact the central nervous system (CNS). Notably, short-chain fatty acids (SCFAs), which are produced through anaerobic fermentation by bacteria in the colon have garnered attention.^[11,12]^ Researchers investigating SCFAs, have posited a broad-ranging impact on CNS function. Alterations in the composition of microbial metabolites, including SCFAs, have been observed in the bodies of individuals with PD.^[13]^ Nevertheless, it is noteworthy that, although SCFA concentrations in the plasma of patients with PD show an increase, levels in fecal matter exhibit a decreasing trend. This phenomenon may be linked to the severity of PD, intestinal permeability, or alterations in the gut microbiota.^[14,15]^ Despite a substantial body of evidence demonstrating the influence of SCFAs on PD, the specific mechanisms remain elusive. Some studies suggest that the effects of SCFAs on PD may be mediated through the regulation of neuronal epigenetics.^[16]^ Additionally, research indicates that these fatty acids may impact PD by modulating immune inflammation in the human body.^[17]^ Consequently, exploring the mechanisms of SCFA action is imperative for advancing our understanding of the pathogenesis of PD.

This article provides a comprehensive review of the primary mechanisms by which SCFAs impact PD. First, we summarize the synthesis, metabolism, and related functions of SCFAs, elucidating the mechanistic pathways involving SCFA-mediated G protein-coupled receptors and the inhibitory effects of SCFAs on histone deacetylation. Second, in conjunction with the intestinal microbiota, we delineate the connection between SCFAs and PD. Subsequently, we analyze the inflammatory response, oxidative stress, abnormal aggregation of α-Syn, and mechanisms associated with the gut-brain axis instigated by SCFAs in PD. Finally, we explore the prospects of utilizing SCFAs as a therapeutic target for treating PD.

## 2. Methods

We conducted an exhaustive literature search in PubMed and Web of Science databases through 2024. The combination of search terms used included (“intestinal microbe” OR “intestinal microflora” OR “intestinal microbiota” OR “gut microbe*” OR “intestinal microorganism*” OR “gut microbiome*”) AND (“short chain fatty acid*” OR “short-chain aliphatic acid” OR “volatile fatty acids”) AND (“Parkinson disease” OR “Parkinson’s disease”), (“short chain fatty acid*” OR “short-chain aliphatic acid” OR “volatile fatty acids”) AND (“G protein-coupled receptor*” OR “G protein coupling receptors” OR “G-coupled protein receptor”), (“short chain fatty acid*” OR “short-chain aliphatic acid” OR “volatile fatty acids”) AND (“histone deacetylase*”), (“short chain fatty acid*” OR “short-chain aliphatic acid” OR “volatile fatty acids”) AND (“inflammatory response” OR “inflammation” OR “oxidative stress” OR “oxidant stress” OR “oxidation stress” OR “oxidative stress induced” OR “α-Synuclein” OR “gut-brain axis” was designed to capture all relevant research literature. Our inclusion criteria were set as peer-reviewed research papers published between 2019 and 2024, and the language of the article had to be English. Exclusion criteria included case reports and conference abstracts.

By reading the titles and abstracts, literatures that were not relevant to the research topic were excluded. The remaining literatures were then reviewed in full text. To ensure the quality of the literature, we only include articles from the Science Citation Index Expanded.

## 3. Mechanisms of action of SCFAs

### 3.1. Synthesis, metabolism, and fundamental functional aspects of SCFAs

SCFAs are among the most extensively studied metabolites originating from the gut microbiota. SCFAs, which consist of saturated fatty acids containing 1 to 6 carbon atoms, manifest in diverse forms, with acetic (C2), propionic (C3), and butyric acid (C4) prevailing in a proportionate distribution of 60:20:20 within the intestinal milieu.^[18–20]^ The biosynthesis of SCFAs by gut microbes is a multifaceted and intricate process. Gut microbiota initiate the breakdown of undigested dietary fibers within the body through exogenous enzymes.^[21]^ Subsequently, these microbiota ferment the resulting oligosaccharides from fiber degradation,^[22]^ fostering the generation of SCFAs through metabolic activities within the gut microbial community, including genera such as Bacteroides and Prevotella.^[11]^ However, studies indicate that, although Prevotella demonstrates a proportional relationship with acetate production, its impact on yields of propionate and butyrate remains somewhat ambiguous,^[23]^ underscoring the collaborative and interactive influence of gut microbial consortia in the production of these metabolites. Additionally, a minor fraction of SCFAs is derived from the metabolism of certain proteins and peptides within the gastrointestinal tract by clostridia,^[24]^ and probiotics such as Lactobacillus and Bifidobacterium within the gut environment also engage in SCFA production through lactic acid fermentation.^[25]^

**Figure 1.**
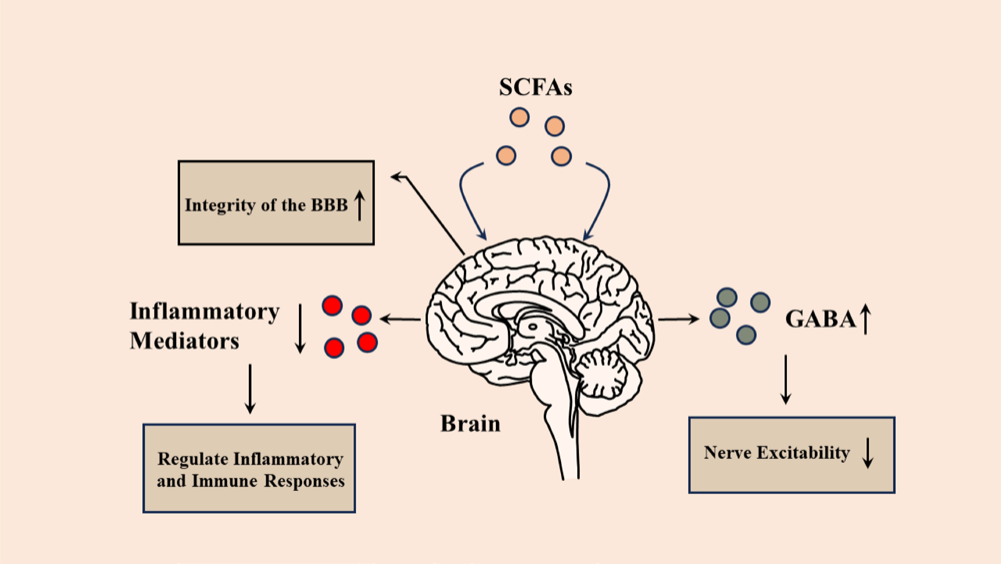
Functional Expression of Short-Chain Fatty Acids in the Central Nervous System. In the CNS, SCFAs not only significantly enhance the integrity of the BBB by reducing inflammatory mediators to regulate immune and inflammatory responses but also increase GABA synthesis and decrease neuroexcitability in patients with PD. BBB = blood-brain barrier, CNS = central nervous system, GABA = γ-aminobutyric acid, PD = Parkinson disease, SCFAs = short-chain fatty acids.

Within a low-oxygen environment, gut microbiota engage in the decomposition of organic substances to produce SCFAs as an energy source, subsequently, these SCFAs are absorbed by intestinal cells, where they play pivotal roles in metabolic and physiological functions within the body as a source of energy for the intestinal cells.^[26]^ Approximately 95% of SCFAs are absorbed by the colon and rectum. The epithelial cells in these absorption sites are equipped with specialized transport systems, such as sodium-coupled monocarboxylate transporter 1 and hydrogen-dependent monocarboxylate transporter (MCT), significantly enhancing the efficiency of SCFA absorption.^[27,28]^ Following absorption, SCFAs manifest a multitude of functions, which include providing nutrition, maintaining intestinal mucosal health, regulating immune function, and influencing energy metabolism.^[29–31]^ SCFAs can impact the structure and function of the CNS through various pathways. Studies have suggested that SCFAs can enhance the integrity of the blood–brain barrier (BBB) and play a crucial role in regulating inflammatory and immune responses.^[32,33]^ Moreover, SCFAs affect neurotransmitters and neural function. For example, butyrate can increase the synthesis and release of inhibitory neurotransmitters such as γ-aminobutyric acid (GABA), thereby modulating the balance between CNS excitation and inhibition.^[34]^ This may have implications for non-motor symptoms in PD, underscoring the importance of the gut microbiota community and its metabolic byproducts in maintaining ecological stability within the human body. The functions of SCFAs in CNS are shown in Figure 1.

### 3.2. Involvement of SCFAs in signal transduction

#### 3.2.1. Multiple receptors mediated by SCFAs

After years of extensive research, it has been established that SCFAs serve as mediators for multiple signaling receptors. Among these, extensively studied receptors include GPR43/free fatty acid receptor 2, GPR41/free fatty acid receptor 3, and GPR109A.^[35,36]^ These transmembrane receptors, which bind to SCFAs, activate signaling pathways through the G protein-mediated cascade. They play crucial roles in regulating various physiological functions such as metabolic regulation, immune responses, and epigenetic control.^[37–39]^ GPR43, the most extensively researched receptor for SCFAs, exhibits a strong affinity for acetic acid, propionic acid, and butyric acid.^[40]^ Upon activation, GPR43 couples with Gαi and Gαq proteins from the Gα subunit family, subsequently stimulating intricate downstream pathways, thereby exerting multifaceted biological effects.^[41]^ GPR43 is predominantly expressed in neutrophils and immune cells, but its presence is notably absent in the brain. Activation of such free fatty acid receptors (FFARs) by SCFAs modulates the activation of immune cells and inflammatory responses in various systems.^[42–44]^ In contrast to GPR43, GPR41 is primarily activated by propionic acid, butyric acid, and valeric acid, and it does not couple with Gαq proteins. GPR41 exhibits widespread expression in the intestine, adipose tissue, and even the CNS.^[45,46]^ Research suggests that GPR41 plays a pivotal role in regulating the sympathetic nervous system and has diverse functions in the CNS.^[47]^ These findings provide a solid foundation for further research into potential therapeutic strategies for PD. GPR109A is mainly expressed in adipose tissue and immune cells. It is activated by butyrate, β-hydroxybutyrate, and niacin.^[48,49]^ Additionally, it has been observed that internalization of the GPR109A receptor can be effectively inhibited by 500 ng/mL of pertussis toxin, indicating that GPR109A also couples with Gαi proteins.^[50]^

In recent years, it has been observed that activation of the mitogen-activated protein kinase (MAPK) pathway is under the regulatory influence of SCFAs, playing a pivotal role in modulating intracellular immune inflammation.^[51]^ Upon activation by SCFAs acting as ligands, G protein–coupled receptors (GPRs) initiate signal transduction. The activated GPRs binds with G proteins, leading to the binding of Gα with GTP and achieving an activated state. Downstream, the activated G protein prompts the phosphorylation of Raf kinase, subsequently initiating the MAPK signaling cascade, including extracellular signal-regulated kinase ½ (ERK1/2), c-Jun NH2-terminal kinase (JNKs), and p38 MAPK.^[52]^ Among these, ERKs are associated with cellular proliferation and differentiation, whereas JNKs and p38 MAPK have been demonstrated to participate in the inflammatory response in the human body.^[53]^ Research suggests that upon binding with its ligand, GPR43 induces the activation of ERK1/2 and p38 MAPK pathways, while the activation of GPR41 primarily mediates the ERK1/2 pathway, and FFARs can stimulate thrombus formation through activation of the JNK pathway.^[54,55]^ Recently, anti-inflammatory effects arising from the inhibition of the MAPK pathway by SCFAs have gained attention. Additionally, there is a growing trend in examining G protein-coupled receptors of this nature, specifically addressing their role in alleviating neuroinflammation by inhibiting the nuclear factor kappa B (NF-κB) pathway.^[56]^ Activation of the NF-κB pathway triggers the expression of a plethora of pro-inflammatory genes, including cytokines, adhesion molecules, and enzymes, which play a crucial role in immune inflammatory responses.^[57]^ Reports suggest that SCFAs can mediate activation of the GPR43 receptor, inhibiting the NF-κB pathway, to regulate inflammatory responses.^[58]^ Furthermore, following activation by niacin, GPR109A has been found to possess an inhibitory effect on the phosphorylation and nuclear translocation of NF-κB protein (p-NF-κB), thereby mitigating neuroinflammation, particularly in conditions such as PD.^[59]^ Despite the potential impact of interaction between SCFAs and G protein-coupled receptors on neurodegenerative diseases, research in this area remains relatively limited, necessitating a substantial influx of knowledge to fill existing gaps.

#### 3.2.2. SCFAs suppress histone deacetylation

Existing research has substantiated the prominent role of epigenetic regulation in the pathogenic mechanisms of PD, with histone modification being a pivotal facet of epigenetic control intricately linked to PD.^[60]^ Histone modification encompasses acetylation, methylation, phosphorylation, and ubiquitination. Among these, histone acetylation stands out as a critical epigenetic mechanism in PD, offering an exploratory avenue to improve genetic understanding regarding the onset and progression of PD.^[61,62]^ Histone acetylation is primarily orchestrated by the dynamic regulation of histone acetyltransferases (HATs) and histone deacetylases (HDACs). HATs selectively transfer acetyl groups to lysine residues on the histone tail, resulting in chromatin relaxation. This alteration facilitates the specific binding of various transcription factors, promoting gene transcription. Conversely, HDACs remove these acetyl groups, inducing changes in chromatin structure and gene expression. This process leads to the tight packaging of chromatin, rendering genes less amenable to transcription.^[63,64]^ The HDACs family comprises Class I, Class II, Class III (also known as Sirtuins, SIRT1-7), and Class IV. Class I, Class II, and Class IV HDACs are zinc-dependent, whereas Sirtuins are dependent on nicotinamide adenine dinucleotide (NAD+).^[65]^

Due to the mechanism of gene transcription inhibition by HDACs, certain HDACs, particularly those of Class I, can induce reduced expression of the a α-Syn encoding gene (SNCA gene).^[66]^ This phenomenon, in turn, affects the production of α-Syn, leading to decrease aberrant aggregation of α-Syn and an reduced risk of PD.^[67]^ Additional research indicates that HDACs influence the stability of leucine-rich repeat kinase 2 (LRRK2) protein, which is rich in leucine residues, through various pathways, including transcription and post-translational modifications. Dysregulation of LRRK2, thus induced, is closely associated with neuronal damage and death, particularly in the genetic forms of PD.^[68]^ The inhibitory effect of HDACs on the NF-κB pathway is also noteworthy. HDACs inhibit NF-κB nuclear translocation, preventing their entry into the nucleus and activation of the NF-κB pathway, consequently, imparts a certain degree of anti-inflammatory action.^[69]^ However, the specific mechanistic impact of HDACs on PD remains unclear, and whether it directly leads to the death of dopamine neurons or follows alternative pathways necessitates further in-depth investigation.

**Figure 2.**
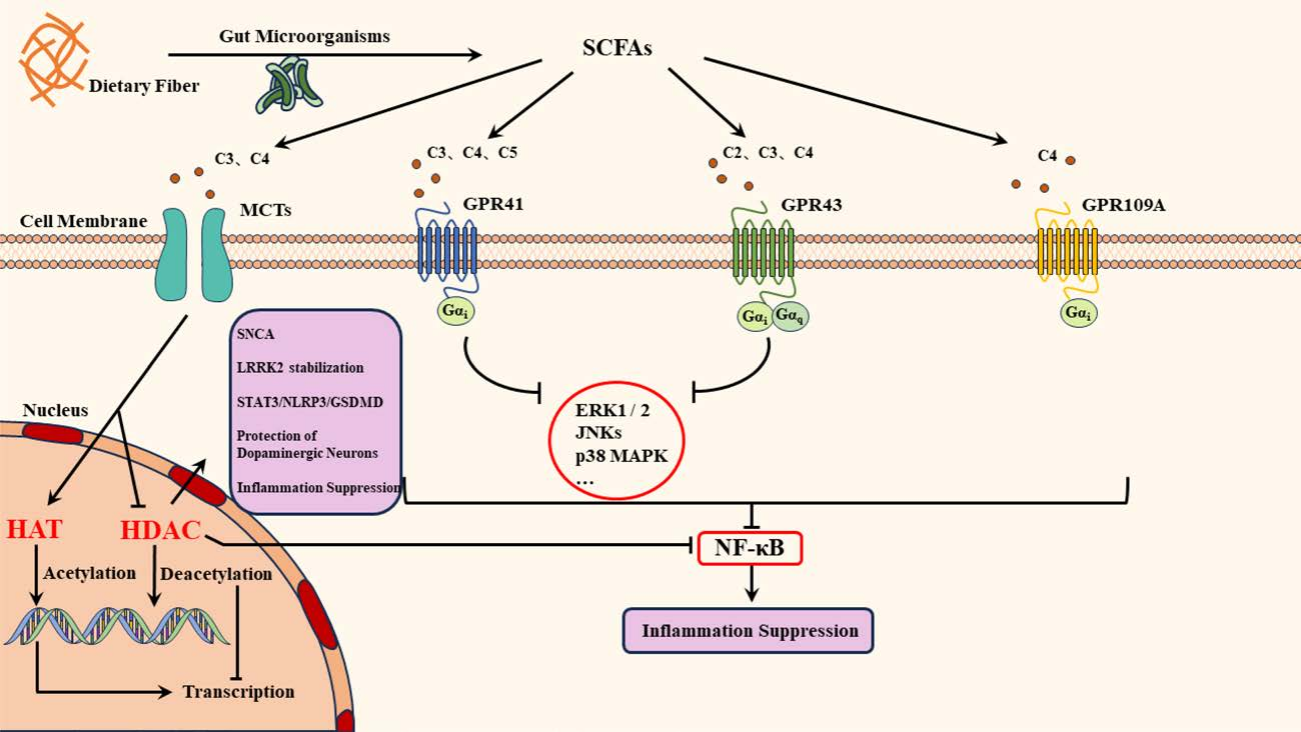
Cellular Signal Transduction Pathways of Short-Chain Fatty Acids. SCFAs exhibit dual mechanisms in mediating intracellular anti-inflammatory responses. Through the activation of G protein-coupled receptors such as GPR41, GPR43, and GPR109A, SCFAs orchestrate downstream signaling cascades involving NF-κB and MAPKs pathways. Simultaneously, these bioactive compounds traverse cellular membranes via transport systems, notably MCTs, facilitating their entry into cells. Once intracellular, SCFAs engage in the intricate regulation of gene transcription by inhibiting HDACs or promoting HATs. Moreover, HDACs can suppress NF-κB nuclear translocation, thereby preventing the activation of the NF-κB pathway. This intricate molecular orchestration gives rise to a myriad of biological effects, contributing to the modulation of various cellular processes. GPR109A = G protein–coupled receptor 109A, GPR41 = G protein–coupled receptor 41, GPR43 = G protein–coupled receptor 43, HATs = histone acetyltransferases., HDACs = histone deacetylases, MAPKs = mitogen-activated protein kinases, MCTs = monocarboxylate transporters, NF-κB = nuclear factor kappa B, SCFAs = short-chain fatty acids.

Histone Deacetylase (HDAC) inhibitors are structurally categorized into hydroxamic acid derivatives, SCFAs, cyclosporin analogs, and aniline derivatives.^[70]^ With effective concentrations in the micromolar range, SCFAs act as broad-spectrum inhibitors effectively preventing histone deacetylation,^[71]^ particularly butyric acid and propionic acid, and exhibiting significant potential for HDAC inhibition. Butyric acid is widely acknowledged as a potent HDAC inhibitor, with an inhibition rate reaching 80%, whereas propionic acid, at ~60%, shows a comparatively lower inhibition rate.^[72]^ Butyric acid and propionic acid can bind to the catalytic center of HDACs, especially butyric acid, whose carboxyl group can form a coordination bond with the zinc ion in zinc-dependent HDACs, leading to structural alterations in the HDAC catalytic center and subsequent loss of activity.^[73]^ As, a subclass of HDACs dependent on NAD+, Sirtuins differ slightly from the others. Research demonstrating the inhibition of SIRT1 by butyric acid suggests that SCFAs can indirectly inhibit HDAC Class III by influencing the NAD+/NADH ratio.^[74]^ Experimental evidence indicates that by, leveraging the inhibitory effects of HDACs in 1-methyl-4-phenylpyridinium (MPP)/1-methyl-4-phenyl-1,2,3,6-tetrahydropyridine (MPTP)-induced PD models, β-hydroxybutyric acid disrupts the STAT3/NLRP3/GSDMD signaling pathway, effectively alleviating clinical and pathological symptoms of PD.^[75]^ Furthermore, post-HDAC inhibition, SCFAs enhance the expression of neurotrophic factors, glial cell-derived neurotrophic factor and brain-derived neurotrophic factor (BDNF) on astrocytes, thereby exerting a protective effect on dopamine neurons.^[76]^ Additionally, studies suggest a reduction in inflammatory responses following HDAC inhibition, which is likely associated with neutrophil apoptosis.^[77]^ The intricate association between histone deacetylation and PD is undeniable; however, the diverse subtypes of HDAC pose significant challenges for therapeutic selection. The signaling mechanisms of SCFAs are illustrated in Figure 2.

## 4. SCFAs and PD

With the aid of various genomics sequencing techniques, it is possible to objectively and effectively analyze the abundance changes of gut microbiota in patients with PD. Over the past few years, research teams have summarized the abundance variability of gut microbial communities involved in SCFA production. Comparative analysis revealed that patients with PD exhibited decreased levels of Prevotella, Bacteroides, Faecalibacterium, and Ruminococcus, among others, compared to the healthy control group. Conversely, species such as Clostridium, Lactobacillus, Escherichia, and Bifidobacterium demonstrate increased abundance in patients with PD.^[78]^ This dysbiosis of gut microbiota might be contributing factor leading to the overall reduction of SCFAs in patients with PD. The close association between SCFAs and PD has garnered considerable attention. In recent years, numerous animal models have robustly demonstrated that supplementation with SCFAs can alleviate clinical symptoms of PD. In the widely used 1-methyl-4-phenyl-1,2,3,6-tetrahydropyridine (MPTP) mouse model, supplementation of butyrate sodium not only relieved PD motor impairments but also effectively inhibited the activation of α-Syn.^[56,79]^ In a 6-hydroxydopamine (6-OHDA) induced mouse model, maintaining gut microbiota homeostasis and increasing propionate levels successfully alleviated motor dysfunction and the loss of dopaminergic neurons.^[80]^ However, it is important to note that most existing animal models primarily measure changes in the SCFA levels in feces. This consideration is necessary because a large quantity of SCFAs are absorbed by the intestines,^[81]^ and the increased gut permeability observed in PD models allows SCFAs to leak into the bloodstream. Therefore, it is advisable to compare the findings with relevant experiments performed in plasma and brain tissues.

There is abundant evidence to suggest that SCFAs are generally beneficial for patients with PD. However, some experiments also indicate that SCFAs can have adverse effects in patients with PD. For example, SCFAs assist in inhibiting the release of inhibitory neurotransmitters and further weaken the inhibitory effects of HDACs on NF-κB, potentially exacerbating inflammatory responses in the nervous system.^[34,69,71]^ Additionally, studies have shown that after oral administration of SCFA mixtures, germ-free (GF) mice experience worsening motor dysfunction and activation of microglial cells.^[82]^ Qiao and colleagues also reported that SCFAs (NaB; 165 mg/kg/d, 7 days) cause substantial harm to MPTP-induced mice, exacerbating key pathological manifestations such as motor impairments and neuroinflammation in PD.^[83]^ These contradictory experimental results further underscore the complex and multifaceted impact of SCFAs on PD. Investigating the specific mechanisms underlying the influence of SCFAs on PD, requires considering, the interplay of other factors within the body.

### 4.1. SCFAs and inflammatory responses

In recent years, there have been reports indicating that patients with mutations in the PINK1 gene (a key pathogenic gene for PD) exhibit a distinctive increase in interleukin-6 (IL-6) levels.^[84]^ Furthermore, a plethora of evidence suggests a significant elevation in pro-inflammatory cytokines and chemokines in patients with PD, including tumor necrosis factor alpha (TNF-α), IL-1β, IL-10, calprotectin, C-reactive protein (CRP), and interferon gamma (IFN-γ).^[85–88]^ The upregulation of these pro-inflammatory factors stems from various factors, primarily through the toll-like receptor 4 (TLR4) pathway. TLR4 specifically recognizes signals of endogenous and exogenous damage, predominantly lipopolysaccharides (LPS) generated by intestinal bacteria in PD. Upon stimulation, TLR4 activates the MyD88 pathway, subsequently triggering NF-κB release and the secretion of a plethora of inflammatory mediators.^[89]^ In the brain, the aggregation of aberrant α-Syn and preexisting neuronal damage can also stimulate TLR4, initiating the activation of microglia and astrocytes, leading to the release of substantial pro-inflammatory factors.^[90]^ Studies using a TLR4-knockout PD model combined with oral administration of fisetin have revealed a significant attenuation of PD-like pathological manifestations, emphasizing the crucial role of TLR4 in PD pathology.^[91]^ These inflammatory factors not only induce peripheral inflammation but also elicit intestinal inflammation. Moreover, they can traverse the BBB, resulting in neuroinflammation, thereby contributing to oxidative stress and other PD-like pathological responses.^[92,93]^ This interplay not only impacts the progression of PD but also provides novel insights for therapeutic interventions.

The primary battleground for neuroinflammation is microglia and astrocytes. Under normal circumstances, microglia are widely distributed in the brain, maintaining the normal immune function and stable development of the CNS, akin to macrophages, to protect the CNS from external pathogens.^[94]^ In the PD microenvironment, aggregated α-Syn promotes the transition of microglia from a resting state (M0) to an activated pro-inflammatory state (M1), leading to a rapid increase in pro-inflammatory cytokines and exacerbating neuroinflammation.^[95]^ In contrast, SCFAs shift microglia from the M1 to the M2 phenotype, thereby resisting neuroinflammation.^[96]^ Current research indicates a regulatory role of SCFAs on microglia, with GF mice lacking SCFA regulation and exhibiting noticeable developmental abnormalities in microglia.^[97]^ In in vitro experiments show that the combined use of acetate and butyrate is more effective in suppressing inflammatory factor production in LPS-stimulated microglia than treatment with individual SCFAs.^[98]^ In an alcohol-induced neuroinflammation model induced the expression of peroxisome proliferator-activated receptor-gamma through coupling with the GPR109A receptor, inhibiting activation of the TLR4-NF-κB pathway and thereby suppressing damage to the CNS by microglia.^[99]^ Furthermore, under inflammatory stimulation, astrocytes transition from a resting state to an A1 reactive state, releasing inflammatory factors. Astrocytes play a crucial role in maintaining BBB function and providing nutritional support to neurons. The inhibitory effect of SCFAs on microglia has a positive feedback impact on astrocytes.^[94]^ Interestingly, the sex-specific effects of SCFAs on astrocytes have been observed, with sodium butyrate influencing the expression of BDNF and Pgc1-α only in female individuals, which is probably associated with the inhibition of HDAC activation.^[100]^ Additionally, SCFAs can stimulate the expression of the odor receptor Olfr920 in astrocytes, reducing their activity.^[101]^ However, the specific mechanisms by which SCFAs influence neuroinflammation remain unclear due to variations in experimental models and research methodologies.

### 4.2. SCFAs and oxidative stress

In daily life, the mitochondria within eukaryotic cells predominantly supply energy to the human body through oxidative phosphorylation, generating adenosine triphosphate (ATP) concomitantly with the production of reactive oxygen species (ROS). These ROS include superoxide anions hydrogen peroxide and secondary byproducts such as hydroxyl radicals. Approximately 90% of ROS originate from mitochondrial oxidative phosphorylation.^[102]^ Although normal levels of ROS are essential to maintaining cellular physiological functions, dysfunction in mitochondrial processes, iron accumulation, and inflammatory responses collectively contribute to an excessive accumulation of ROS. This disrupts the redox balance within cells, leading to oxidative stress and irreversible cellular damage.^[92,103,104]^ Given the heightened activity of neurons in the human body, mitochondrial dysfunction and oxidative stress emerge as pivotal contributing factors to PD. Commonly used induction mechanisms in PD animal models involve oxidative stress-induced damage to dopaminergic neurons in the substantia nigra.

The relationship between SCFAs and oxidative stress has been an active area of research. In particular, the significant regulatory role of butyric acid and propionic acid in the Keap1-Nrf2 system in oxidative stress cannot be overlooked. Through the Keap1-Nrf2-ARE pathway, SCFAs exert a protective effect against oxidative stress.^[105]^ Nuclear factor erythroid2-related factor 2 (Nrf2), a primary regulatory factor, encodes numerous antioxidant enzyme genes in the human body.^[106]^ Typically, Nrf2 binds to the inhibitory factor Keap1. Under oxidative stress conditions, these dissociate, allowing Nrf2 to translocate to the nucleus, bind to antioxidant response elements and activate the expression of antioxidant enzymes such as superoxide dismutase (SOD), glutathione peroxidase (GPx), glutathione S-transferase, and uridine diphosphate glucuronosyltransferase.^[107]^ Extensive animal experiments corroborate the significant impact of SCFAs on alleviating oxidative stress. In a PD model induced by rotenone, the addition of 40 mg/kg butyric acid markedly increased antioxidant enzyme levels,^[108]^ and propionic acid substantially reduced reactive oxygen species production in dopamine neurons induced by 6-OHDA.^[109]^ Abundant evidence suggests that butyrate salt, coupled with GPR43, alleviates high glucose-induced oxidative stress, involving the inhibition of HDACs and the NF-κB pathway, showcasing a multifaceted cooperative result.^[58]^ In vitro experiments show that the addition of butyrate salt enhances antioxidant enzyme activity,^[110]^ which is possibly associated with HDAC inhibition. Furthermore, researchers introduced butyrate salt into MPTP-induced animal models and MPP-induced in vitro PC12 cell models, discovering its ability to reduce neuroinflammation and oxidative stress damage to neurons by inhibiting the JAK2/STAT3 signaling pathway.^[111]^ However, evidence also suggests that acetate can promote the release of reactive oxygen species within neutrophils,^[112]^ challenging the notion that SCFAs rescue oxidative stress. This indicates that the specific mechanisms by which SCFAs influence oxidative stress require further investigation, emphasizing the complexity that researchers must consider to understand their relationship.

### 4.3. SCFAs and aberrant aggregation of α-Synuclein (α-Syn)

Since researchers elucidated the aggregation of α-Syn into Lewy bodies, thereby establishing its association with PD, the transgenic α-Syn overexpression (ASO) model has emerged as a consequential paradigm for investigating the role of α-Syn in PD.^[113]^ Within the healthy brain, α-Synuclein is present in neurons, actively participating in the release of signal molecules such as dopamine within synaptic vesicles.^[114]^ Under the influence of such factors as inflammation, oxidative stress, and mutations in the SNCA gene, α-Syn undergoes a transition from its soluble α-helical structure to insoluble β-folded conformations. This transformation leads to abnormal aggregation, giving rise to Lewy bodies in the dopamine neurons of the substantia nigra in the brain.^[67,92,115]^ Consequently, this process induces dysfunction and death in dopaminergic neurons, leading to the depletion of dopamine, a pivotal factor in the manifestation of motor dysfunction observed in PD. Simultaneously, the aberrant aggregation of α-Syn also triggers neuroinflammation and mitochondrial damage,^[116,117]^ indicating an interplay of various pathological manifestations in PD, forming a vicious cycle. Notably, aggregated α-Syn in the brain can propagate in a prion-like manner within the body, emerging as a crucial factor in the pathological progression of PD.^[118]^ Recent studies suggest that in ASO mouse models, a diet enriched in high-fiber prebiotics can reduce α-Syn aggregation. This dietary intervention effectively protects microglial cells, mitigating neuroinflammation. Furthermore, the downregulation of α-Syn aggregation by SCFAs can be achieved indirectly through the inhibition of oxidative stress and neuroinflammation.^[115,119]^ In patients with PD, α-Syn is detected in both the CNS and the enteric nervous system (ENS). A team investigating the relationship between α-Syn in enteroendocrine cells and sodium butyrate found that sodium butyrate activates the expression of autophagy gene Atg5 while inhibiting the PI3K/Akt/mTOR pathway to mediate the degradation of α-Syn.^[120]^ Additionally, in a rotenone-induced model, sodium butyrate can initiate autophagy by promoting the expression of PGC-1α, facilitating the degradation of α-Syn.^[121]^ Understanding the precise mechanisms underlying the interaction between SCFAs and α-Syn holds promise for unveiling novel therapeutic strategies for PD.

### 4.4. SCFAs and the gut brain axis

In 2006, Braak et al put forth the hypothesis that PD may originate from the gastrointestinal tract based on the deposition pattern of α-Syn. Experimental findings indicated that early PD changes emanate from the olfactory bulb and dorsal root ganglia of the vagus nerve. Subsequently, α-Syn deposition extends to the brain, particularly evident in postmortem examinations of PD patients, where researchers identified α-Syn deposits in the intestines, further substantiating the crucial pathway connecting the gut and the brain.^[122]^ Concurrently, studies suggest that vagus nerve severance confers a protective effect in patients with PD, with the vagus nerve considered a primary conduit for this pathway.^[123]^ After years of investigation, understanding of the intricate interplay between the gut, gut microbiota, and the brain has matured, underscoring its pivotal importance in PD research.^[124]^ The gut brain axis constitutes a complex bidirectional communication system involving the ENS, the autonomic, and the CNS.^[125]^ The ENS, often referred to as the “second brain,” plays a crucial role in the interaction between the gut microbial community and the CNS within the gastrointestinal tract. Unger and colleagues postulated that microbial populations capable of producing SCFAs in the intestines are implicated in the formation of Lewy bodies in the ENS, and alterations in the ENS are regulated by SCFAs.^[126]^ The impact of SCFAs on the ENS is multifaceted. Comparative studies using rodent models fed a resistant starch or versus standard diet revealed that butyrate can modulate ENS neuron excitability through coupling with GPR41.^[127]^ SCFAs also influence the release of serotonin (5-HT) and GABA, thereby affecting intestinal motility and ENS function.^[128]^ Furthermore, butyrate enhances the expression of tight junction proteins, maintaining intestinal barrier function, preventing harmful substances from entering the intestinal lumen, and concurrently establishing a close connection with the gut brain axis to maintain homeostasis.^[129]^ The impact of SCFAs on ENS function consequently influences the expression of the gut brain axis, exerting an effect on the onset and progression of PD.

Simultaneously, the BBB plays a pivotal role in safeguarding the integrity of the gut brain axis. Through observation of GF mouse BBB processes, Braniste and colleagues noted a significant loss of tight junction proteins and increased BBB permeability. Symptoms improved upon colonization with normal mouse microbiota and SCFAs, highlighting the regulatory capacity of SCFAs on the BBB.^[130]^ Numerous studies confirm that SCFAs can traverse the BBB and influence its function and integrity. SCFAs serve as an energy source for endothelial cells in the brain, repairing mitochondrial damage caused by oxidative stress and maintaining normal endothelial cell function.^[131]^ Additionally, by inhibiting HDAC activity, butyrate increases the expression of tight junction proteins such as occludin and claudin-5, thereby enhancing the structural integrity of the BBB.^[132]^ G protein-coupled receptors, particularly GPR41 and GPR109A, widely expressed in brain endothelial cells, play a significant role in the inhibitory regulation of neuroinflammation and protection of structural integrity by SCFAs.^[32]^ Moreover, physiological concentrations of propionate can protect the BBB from oxidative stress-induced damage through the Nrf2 pathway.^[133]^ Although the BBB is closely associated with the gut brain axis, the specific mechanisms through which SCFAs influence the gut brain axis via the BBB require more in-depth investigation in basic research and clinical trials. The causal relationship between the gut brain axis and SCFAs remains unclear, necessitating further research to establish whether changes in SCFAs lead to alterations in the gut brain axis.

## 5. Therapeutic implications of SCFAs for PD management

In recent years, the global scientific community has increasingly recognized SCFAs as a novel therapeutic avenue for PD, sparking significant enthusiasm for extensive research. Investigations have focused on strategies such as supplementing SCFAs or restoring gut microbiota stability to foster the production of SCFAs, aiming to achieve therapeutic efficacy. Notably, studies have indicated a postprandial reduction in glucagon-like peptide-1 levels in patients with PD, and sodium butyrate has emerged as a potent therapeutic agent capable of upregulating glucagon-like peptide-1 levels, thereby enhancing its neuroprotective effects on the CNS.^[134,135]^ Additionally, research teams have integrated sodium butyrate into probiotics, demonstrating a synergistic effect in restoring neurodegeneration and preventing neuronal damage. This combined approach has shown promise in treating nerve function impairment in MPTP-induced mice and rotenone-induced mice.^[136]^ Similarly, in an in vitro model induced by rotenone, the use of fasudil with the addition of propionic acid exhibited therapeutic potential for dopaminergic neurons, although the specific mechanism remains unclear.^[137]^ Several strategies for modulating SCFA levels in patients with PD have been proposed. These include oral or intravenous supplementation of SCFAs to address deficiencies in patients with PD. However, the drawback of oral administration lies in the unavoidable intestinal absorption of SCFAs, may be influenced by various factors including gastrointestinal symptoms in patients with PD, dysbiosis of the intestinal microbiota, or drug interference, thereby affecting their absorption rate.^[138–140]^ Furthermore, sustained or periodic intravenous infusion of SCFAs may lead to side effects such as venous inflammation.^[141]^ Another approach involves enhancing dietary patterns, such as structuring a high dietary fiber intake, to promote anaerobic fermentation of dietary fiber by gut microbiota and generate SCFAs. High dietary fiber intake has the additional benefit of alleviating constipation symptoms, a common non-motor symptom of PD.^[142]^ A team compared the dietary habits of PD patients with an average disease duration of 9 years to those of the general population and found that due to factors such as non-motor functional impairments and medication use, the former group tends towards a diet high in carbohydrates and fats, lacking in high-quality, fiber-rich foods that could alleviate constipation and regulate the gut microbiome. This inclination towards a lower quality diet is detrimental to the progression of the disease.^[143]^ Presently, fecal microbiota transplantation (FMT) dominates the landscape of PD treatment strategies. FMT holds the potential to comprehensively reconstruct the gut microbiota, maintaining microbial homeostasis, and increasing SCFA levels through microbial resettlement.^[144]^ Moreover, research indicates that FMT can downregulate TLR4/TNF-α signaling pathway expression in both the gut and the brain.^[145]^ However, despite theoretical and experimental support, the clinical application of FMT faces numerous challenges, including patient acceptance, potential side effects, and production costs.^[146]^ Future research endeavors are warranted to comprehensively address these issues.

## 6. Conclusion

In summary, as a primary metabolic byproduct of intestinal microbiota, the impact of SCFAs on PD cannot be overlooked. Continuously accumulating evidence suggests a remarkably intricate and complex relationship between SCFAs and PD. However, the potential of aberrant alterations in SCFAs as a causative factor for PD remains a subject requiring further investigation. This paper elucidates the mechanistic actions of SCFAs, revealing their predominant role in influencing the pathological progression of PD through modulating a cascade of signal transduction pathways. By altering the deacetylation of histone proteins involved in epigenetic regulation, SCFAs contribute to several biological effects, including resistance against neuroinflammation, alleviation of oxidative stress, degradation of α-Syn aggregates, and protection of the gut brain axis, comprising a series of beneficial effects in the context of PD. Nevertheless, it is crucial to not disregard potential exacerbation of the PD process by SCFAs. The progression of PD involves multiple systems and tissues, necessitating a multifaceted consideration of how SCFAs affect this intricate process. Additionally, attention must be directed toward the diverse types, concentrations, and cellular environments of SCFAs reported in various studies. Finding the equilibrium point between physiological concentrations of SCFAs and optimal experimental concentrations is paramount for advancing PD research. Extensive research indicates that SCFAs hold promise as a therapeutic avenue in PD. However, it is imperative to note that most studies are based on short-term effects. The question of whether SCFAs can exert long-term influences on PD remains unclear. Clinical trials, based on findings from animal models, are essential to observe the sustained impact of SCFAs on the progression of PD and the long-term quality of life for patients.

## Acknowledgments

We thank LetPub (www.letpub.com) for its linguistic assistance during the preparation of this manuscript, and we would like to express our gratitude to MOTIFOLIO (www.motifolio.com) for providing the brain materials for Figure 1.

## Author contributions

**Conceptualization:** Jiaji Liu, Ruijun Su.

**Investigation:** Jiaji Liu, Qi Chen, Ruijun Su.

**Methodology:** Jiaji Liu, Ruijun Su.

**Writing – original draft:** Jiaji Liu.

**Writing – review & editing:** Ruijun Su.
